# Men and Animals

**Published:** 1882-03

**Authors:** 


### HALL'S JOURNAL OF HEALTH

AND

### MISCELLANY.

MONTHLY.

E. H. GIBBS, A. M., M. D., EDITOR.

FOUNDED IN 1854.

### MEN AND ANIMALS.

WHEN a man realizes that his body is made from the same materials that compose the bodies of all other creatures; that he is subject to the same general laws that govern them; in fact, that he is an animal, he has comprehended an important fact, and if wise, has learned a useful lesson. The same conditions that favor the soundest health and best development and greatest usefulness of the horse apply with even greater force to man, since his body is the house in which his spirit dwells, and his usefulness depends mainly on its condition. There are certain breeds of domestic animals so far superior to the original stock from which they sprang as to seem to belong to another race. The reason of this superior development and increased value is no secret. It has come through better feeding and more careful attention to the general comfort and freedom from hardships of the young animals, through several generations, each generation being an improvement on the preceding one until perfection is attained. Deterioration of races comes inevitably from the opposite course. Soon after the discovery of America, the finest breeds of horses from Europe and Asia were imported into this country. Their progeny is the mustang and the Indian pony; dwarfed to their present dimensions through hardships imposed on them by the most cruel of all people who pretend to be civilized. Feeding has a wonderful influence on the dispositions of men and animals. People who are not well-nourished are always irritable. The half-famished mustang "bucks," kicks and bites. Large men of full habit are always cheerful and happy. Slender persons, whose leanness is due to lack of substantial food, are always fretful and unhappy. The natural inference from these premises is that the main-spring of a man's disposition is located in the stomach; and in a great measure this is true. But let no one fall into the error of thinking that I advocate gluttony. The glutton is not only an animal, but one of low order.

Moderation in all things is a golden rule. The amount of fuel required to run an engine depends on its size and the speed required. The same rule applies to foods, for they are fuel. Men and other animals require just enough to supply the waste or combustion that is constantly going on when they rest, and an increased amount when they labor, depending on the severity of the work. Children require more food in proportion to their size than adults, because the organs of the body are more active. They digest their food more rapidly, and hence require feeding oftener. A parent who restricts a child to three meals a day, or who limits the amount of wholesome and nutritious food that a child must have, makes a sad mistake, and one that will be repented of later. The children that usually suffer most from want of sufficient nourishment are the children of the rich, and of the miserably poor. The death rate among children in these extremes of society is probably about the same. The great middle class is the conservator of health, life and energy that each successive generation must depend upon to move "the wheels of commerce" and take the lead in the vast enterprises of modern times.

